# Effectiveness and safety of Chinese herbal medicine formula Gualou Xiebai Banxia (GLXBBX) decoction for the treatment of stable angina pectoris: Protocol for a systematic review

**DOI:** 10.1097/MD.0000000000011680

**Published:** 2018-08-24

**Authors:** Mingtai Chen, Meihuan Li, Lijun Ou, Rongren Kuang, Yingnan Chen, Tao Li, Ling Men, Jian Zhang, Zhong Zhang

**Affiliations:** a Cardiovascular Department, Shenzhen Traditional Chinese Medicine Hospital, Guangzhou University of Chinese Medicine; b Shenzhen Traditional Chinese Medicine Hospital, Guangzhou University of Chinese Medicine, Shenzhen, Guangdong; c Fuwai Hospital Chinese Academy of Medical Sciences, Beijing, China.

**Keywords:** Chinese herbal medicine, Gualou Xiebai Banxia decoction, protocol, stable angina pectoris, systematic review

## Abstract

**Background:**

Stable angina pectoris (SAP) is one of the most common symptoms of coronary heart disease. Chinese herbal medicine (CHM) has been used to treat SAP increasingly due to its less side effects. The subject of this study is to explore the effectiveness and safety of Gualou Xiebai Banxia (GLXBBX) decoction as a kind of CHM for SAP.

**Methods:**

A systematic literature search for articles up to June 2018 will be performed in following electronic databases: PubMed, Embase, the Cochrane Library, China National Knowledge Infrastructure, Chinese Scientific Journals Database, Chinese Biomedical Database, Chinese Biomedical Literature Service System (SinoMed), and Wanfang Database. Inclusion criteria are randomized controlled trials of modified GLXBBX decoction applied on patients with SAP. The primary outcome measures will be coronary heart disease-related clinical evaluation (frequency of acute attack angina, severity of angina pectoris, electrocardiographic changes, and amount of nitroglycerin) and adverse events. RevMan 5.3 software will be used for data synthesis, sensitivity analysis, metaregression, subgroup analysis, and risk of bias assessment. A funnel plot will be developed to evaluate reporting bias and Egger tests will be used to assess funnel plot symmetries. We will use the Grading of Recommendations Assessment, Development and Evaluation system to assess the quality of evidence.

**Results:**

This systematic review study will provide an evidence of GLXBBX decoction for SAP.

**Conclusion:**

The study will give an explicit evidence to evaluate the effectiveness and safety of GLXBBX decoction for SAP.

**Ethics and dissemination:**

This systematic review does not require ethics approval and will be submitted to a peer-reviewed journal.

**PROSPERO registration number:**

CRD 42018094538.

## 1 Introduction

Stable angina pectoris (SAP), which is related to myocardial ischemia by increasing myocardial oxygen demand and reducing diastolic perfusion time, is one of the most common symptom of coronary heart disease (CHD).^[1,2]^ It has been estimated that more than half the patients with CHD were suffering from SAP6, which affect patients’ daily activities and quality of life. Although conventional treatment strategies (revascularization, medication, and lifestyle modification) have been widely applied on SAP patients, there still have been a large number of patients failing to relieve angina-related symptoms completely and having varieties of adverse effects (including dizziness, headache, and nitrate-related tolerance).^[3–6]^ In virtue of shortcomings of conventional treatment strategies above, Chinese herbal medicine (CHM) may provide an alternative and complementary therapy for SAP.

According to the CHM theory, SAP belongs to the CHM domain of “chest pain,” “heartache” majorly caused by “blood stasis,” “phlegm retention,” and “the deficiency in both Yang and Qi.”^[7–10]^ Gualou Xiebai Banxia (GLXBBX) decoction, which is a traditional Chinese herb medicine formula, has been used widely for treating chest pain in China since 25 to 220 AD16. Recently, increasing evidence^[11–19]^ implies that GLXBBX or modified GLXBBX decoction is of benefit to alleviating angina pectoris symptoms and improving the electrocardiogram (ECG) for SAP patients.

There have been numbers of previous clinical researches and reviews about GLXBBX decoction applied on SAP patients; however, the intensity of evidence has been poor and there has been lack of systematic analysis to evaluate the effectiveness and safety of GLXBBX for the treatment of SAP. Only one meta-analysis has been retrieved reporting systematically the effectiveness of GLXBBX decoction applied on the patients with CHD, though the results of which had certain limitations.^[20]^ There were 19 randomized controlled trials (RCTs) included in the meta-analysis by Liu et al.^[1]^ However, the types of angina were unclear, which was of heterogeneity in the types of patients, and the outcomes of CHD-related clinical evaluation were relatively insufficient.

In consideration of the disadvantages of previous studies and incomplete evidences of widespread use of GLXBBX decoction, this systematic review aimed to summarize the effectiveness and safety of GLXBBX decoction in treating SAP patients.

## 2 Methods and analysis

### 2.1 Registration

The study protocol has been registered on international prospective register of systematic review (PROSPERO). The trial registration number of PROSPERO is CRD 42018094538. The procedure of this protocol will be conducted according to the Preferred Reporting Items for Systematic Review and Meta-analysis Protocols (PRISMA-P) guidance.^[21]^

### 2.2 Eligibility criteria

#### 2.2.1 Type of study

We will include all the RCTs that investigated the effectiveness and safety of modified GLXBBX decoction combined with pharmacotherapy for the treatment of SAP.

#### 2.2.2 Participants

The study will include patients diagnosed as SAP regardless of their age, sex, ethnicity, education, or economic status and whether or not they were outpatients or inpatients. The diagnostic criteria of SAP are as follows.

The diagnostic criteria of CHD should be confirmed according to one of the past or current definitions. The diagnostic criteria include “Nomenclature and criteria for diagnosis of ischemic heart disease”^[22]^ or “ACC/AHA 2002 guideline update for the management of patients with chronic stable angina task force on practice guidelines (committee to update the 1999 guidelines)”^[23]^ or “Practice of internal medicine.”^[24]^

### 2.3 Interventions

Interventions involving the combination of modified GLXBBX decoction with conventional pharmacotherapy are eligible in intervention group. The same conventional pharmacotherapy must be used in the control group.

### 2.4 Outcome

The primary outcome measures will include: CHD-related clinical evaluation (frequency of acute attack angina, severity of angina pectoris, electrocardiograph's change, and amount of nitroglycerin) and adverse events. The secondary outcome measures will include: levels of total cholesterol, triglyceride, low-density lipoprotein cholesterol, and high-density lipoprotein cholesterol levels; Traditional Chinese Medicine syndrome.

### 2.5 Search strategy

The following electronic bibliographic databases will be searched from inception to June, 2018: PubMed, Embase, the Cochrane Library, China National Knowledge Infrastructure, Chinese Scientific Journals Database, Chinese Biomedical Database, Chinese Biomedical Literature Service System (SinoMed), and Wanfang Database. There are no limits on the language of publication. Only clinical trials as a limitation will be included and searched. The following sources will also be searched to identify clinical trials, which are in progress or completed: Clinical Trials.gov and World Health Organization clinical trials registry. The additional relevant studies will also be retrieved from the reference lists of systematic reviews and included studies. We will map search terms to controlled vocabulary if possible. In addition, the search strategy for selecting the fields of title, abstract or keyword will be different referring to the characteristics of databases. Search terms are grouped into 3 blocks (see Table 1).

**Table 1:**
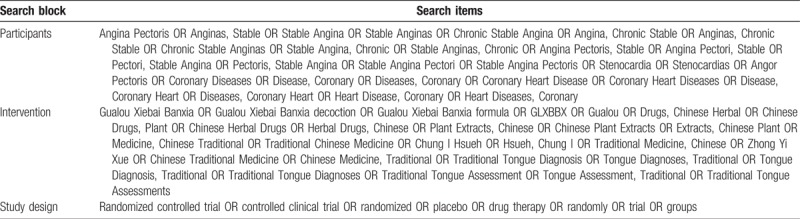
Search items.

### 2.6 Study selection and data extraction

**Figure 1:**
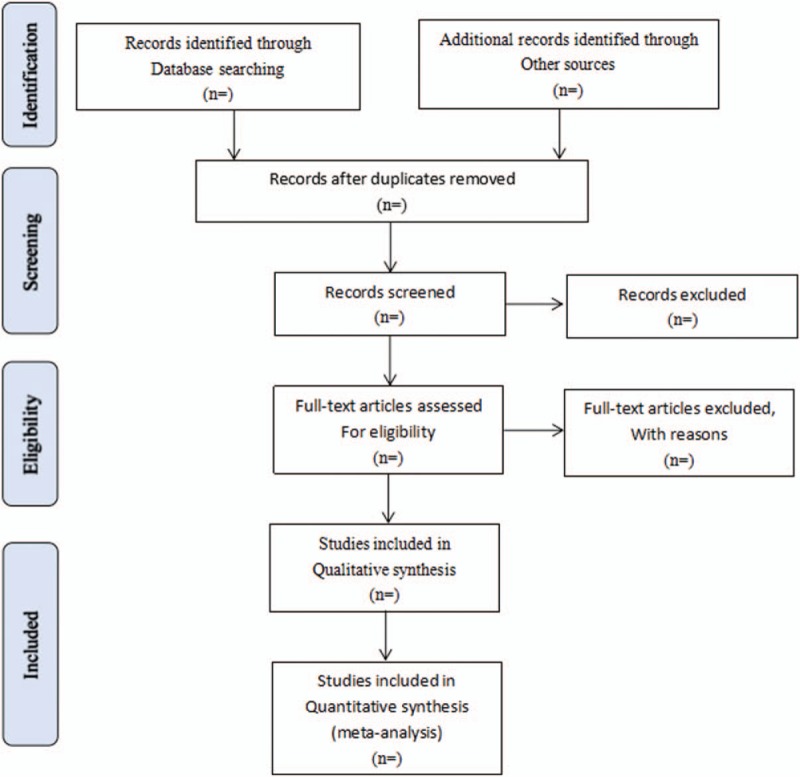
Flow diagram of study selection process. PubMed, Embase, the Cochrane Library, CNKI, VIP Database, CBM, SinoMed, and Wanfang Database.

Literature retrieved citations will be managed by EndNote X7 software. Two authors (MC and LM) will screen the titles and abstracts of the all studies retrieved in above electronic databases independently to find potentially eligible studies. Articles which are duplicated or not accordant with eligibility criteria, intervention and outcome in this study will be exclude. After filtering the final eligible articles, the data from the included articles will be extracted independently from 2 authors (MC and LM). Disagreements will be resolved by discussion or arbitrated by a third author if needed. The following data items will be extracted: first author, publication year, diagnose information, age, sex, trial characteristics, interventions and controls, participants, study methodology, outcomes, adverse events, etc. (see Fig. 1).

### 2.7 Risk of bias assessment

The methodological quality of the eligible studies will be evaluated according to the Cochrane Collaboration's tool for assessing risk of bias.^[25]^ The assessment details include: sequence generation, allocation concealment, blinding of participants and personnel, blinding of outcome assessors, incomplete outcome data, selective reporting, and other sources of bias. Each domain will be assessed as “low risk” or “high risk” or “unclear risk” according to the description details of eligible studies.

### 2.8 Data synthesis and statistical analysis

Statistical analyses will be conducted with RevMan 5.3 software provided by Cochrane Collaboration. Data will be presented by risk ratio or odd ratio with its 95% confidence interval (CI) for dichotomous outcomes and standardized mean difference or weighted mean difference with its 95% CI for continuous outcomes. The I^2^ test will be calculated to determine the amount of heterogeneity. The results of the studies could be used the fixed-effect model to combined unless I^2^ statistic is more than 50%, in which cases, the random-effect model will be used.

### 2.9 Sensitivity analysis, subgroup analysis, and meta-regression

If the heterogeneity or inconsistency among the studies was detected, sensitivity analysis or subgroup analysis or meta-regression analysis will be performed. Subgroup analysis will be conducted to explore potential sources of heterogeneity according to the characteristics of studies, including sample size, severity of SAP, dose of CHM formulas, treatment duration, and other relevant parameters. If data extraction is insufficient, we will create a qualitative synthesis.

### 2.10 Publication bias

A funnel plot will be developed to evaluate reporting bias of the included studies. We will use Egger tests to assess funnel plot symmetry and will interpret values of *P* < .1 as showing statistical significance.

### 2.11 Quality of evidence

We will also assess the quality of evidence for the main outcomes with the Grading of Recommendations Assessment, Development and Evaluation approach. The 5 items will be investigated, including limitations in study design, inconsistency, inaccuracies, indirectness, and publication bias.

### 2.12 Patient and public Involvement

Patients and/or public will not involved due to this study belonging to the secondary sources analysis.

## 3 Discussion

GLXBBX decoction has been applied in clinical more than thousands of years. Currently, GLXBBX decoction is commonly and widely used as an alternative therapy for SAP patients in Asia. Preceding studies have showed that GLXBBX decoction contributes to alleviating angina pectoris and improving the ECG for SAP patients.^[12–14]^ Increasing clinical researches reported that GLXBBX decoction has been widely used and of great benefit in improving for SAP patients; however, there has been no complete evaluation of the clinical evidence regarding GLXBBX decoction as intervention for SAP in evidence-based medicine.^[4,7]^

Accordingly, we intend to conduct this systematic review to assess the effectiveness and safety of GLXBBX decoction for SAP patients. The results of this systematic review may help to propose the clinical recommendation for SAP patients and to provide more reliable evidence for GLXBBX decoction's application.

## 4 Ethics and dissemination

This review does not require the ethical approval because there are no concerns about the patients’ privacy. The results of the meta-analysis will be reported according to the PRISMA extension statement and disseminated in a peer-reviewed journal.

## Author contributions

Zhong Zhang and Jian Zhang conceived the study and drafted the protocol. Lijun Ou, Rongren Kuang, Yingnan Chen, and Tao Li revised it. Mingtai Chen, Ling Men, and Meihuan Li developed the search strategies, conducted data collection, and analyzed independently. All authors have approved the final manuscript.

**Conceptualization:** Mingtai Chen, Jian Zhang, Zhong Zhang.

**Data curation:** Mingtai Chen, Ling Men.

**Formal analysis:** Mingtai Chen, Ling Men.

**Funding acquisition:** Zhong Zhang.

**Investigation:** Yingnan Chen.

**Methodology:** Mingtai Chen, Yingnan Chen.

**Project administration:** Yingnan Chen, Jian Zhang, Zhong Zhang.

**Resources:** Zhong Zhang.

**Software:** Mingtai Chen, Meihuan Li.

**Supervision:** Rongren Kuang.

**Validation:** Rongren Kuang, Tao Li, Jian Zhang, Zhong Zhang.

**Visualization:** Mingtai Chen, Meihuan Li.

**Writing – original draft:** Mingtai Chen, Lijun Ou.

**Writing – review and editing:** Mingtai Chen, Lijun Ou, Rongren Kuang, Tao Li.
